# The immune checkpoint TIGIT/CD155 promotes the exhaustion of CD8 + T cells in TNBC through glucose metabolic reprogramming mediated by PI3K/AKT/mTOR signaling

**DOI:** 10.1186/s12964-023-01455-z

**Published:** 2024-01-12

**Authors:** Mingyao Huang, Xiaoqin Yu, Qing Wang, Zirong Jiang, Xiaofen Li, Wei Chen, Chuangui Song

**Affiliations:** 1 Department of Breast Surgery, Clinical Oncology School of Fujian Medical University, Fujian Cancer Hospital, Fuzhou, 350011 China; 2 Department of Breast Surgery, Fujian Medical University Union Hospital, Fuzhou, 350001 China; 3 Department of Thyroid and Breast Surgery, Ningde Municipal Hospital of Ningde Normal University, Ningde, 352100 China; 4 Department of Oncology Surgery, Fujian Provincial Hospital, Fuzhou, 350001 China

**Keywords:** Immune checkpoint, CD155/TIGIT, CD8 + T cells, TNBC, Immunotherapy

## Abstract

**Objective:**

The CD155/TIGIT axis has attracted considerable interest as an emerging immune checkpoint with potential applications in cancer immunotherapy. Our research focused on investigating the role of CD155/TIGIT checkpoints in the progression of triple-negative breast cancer (TNBC).

**Methods:**

We evaluated CD155 and TIGIT expression in TNBC tissues using both immunohistochemistry (IHC) and gene expression profiling. Our experiments, both in vivo and in vitro, provided evidence that inhibiting the CD155/TIGIT pathway reinstates the ability of CD8 + T cells to generate cytokines. To assess the impact of CD155/TIGIT signaling blockade, we utilized Glucose Assay Kits and Lactate Assay Kits to measure alterations in glucose and lactate levels within CD8 + T cells. We employed western blotting (WB) to investigate alterations in glycolytic-related proteins within the PI3K/AKT/mTOR pathways following the inhibition of CD155/TIGIT signaling.

**Results:**

CD155 exhibits heightened expression within TNBC tissues and exhibits a negative correlation with the extent of infiltrating CD8 + T cells. Furthermore, patients with TNBC demonstrate elevated levels of TIGIT expression. Our findings indicate that the interaction between CD155 and TIGIT disrupts the glucose metabolism of CD8 + T cells by suppressing the activation of the PI3K/AKT/mTOR signaling pathway, ultimately leading to the reduced production of cytokines by CD8 + T cells. Both in vivo and in vitro experiments have conclusively demonstrated that the inhibition of CD155/TIGIT interaction reinstates the capacity of CD8 + T cells to generate cytokines. Moreover, in vivo administration of the blocking antibody against TIGIT not only inhibits tumor growth but also augments the functionality of CD8 + T lymphocytes.

**Conclusions:**

Our research findings strongly suggest that CD155/TIGIT represents a promising therapeutic target for treating TNBC.

**Supplementary Information:**

The online version contains supplementary material available at 10.1186/s12964-023-01455-z.

## Introduction

TNBC, a remarkably aggressive form of breast cancer, is characterized by the lack of expression of estrogen receptor (ER), progesterone receptor (PR), and human epidermal growth factor receptor 2 (HER2) [1]. Despite recent advances in therapeutic approaches, TNBC remains challenging to treat due to its heterogeneity and limited targeted treatment options [2]. Over the past few years, there has been a burgeoning fascination with the involvement of immune checkpoint molecules in the progression of cancer [3]. Immune checkpoints are molecules within the immune system capable of eliciting either a positive (costimulatory) or negative (coinhibitory) signal. Therapies involving antibodies targeting these checkpoints have significantly improved clinical outcomes for a range of malignant tumors, including TNBC [4, 5]. Nevertheless, the therapeutic efficacy of anti-PD-L1 mAb and anti-PD-1 mAb remains constrained. PD-1/PD-L1 immune checkpoint inhibitors have shown enhanced overall survival (OS) exclusively in individuals with positive PD-L1 expression, with no statistically significant differences observed in the intention-to-treat population [6]. Hence, it is imperative to explore immunomodulators targeting alternative immune checkpoints for the treatment of malignant tumors.

TIGIT (T cell immunoreceptor with Ig and ITIM domains) is an inhibitory receptor expressed on T cells that shares a ligand, CD155, with the co-receptor CD226 [7]. CD155, also recognized as the poliovirus receptor (PVR), is a transmembrane glycoprotein frequently characterized by elevated expression in several cancer types, including TNBC [8, 9]. Binding of CD155 to TIGIT inhibited T cell activation [10], while binding to CD226 enhanced T cell activation [11]. The presence of TIGIT expression in tumor-infiltrating lymphocytes (TILs) among melanoma patients is associated with tumor metastasis [12]. Furthermore, the presence of TIGIT expression on CD8 + T cells within peripheral blood mononuclear cells (PBMCs) obtained from gastric cancer patients is correlated with decreased overall survival [13]. Increasing evidence indicates that the CD155/TIGIT signaling pathway assumes a pivotal role in modulating the immune microenvironment across various cancer types [14, 15]. Nonetheless, the mechanisms responsible for CD155/TIGIT-induced immune suppression and evasion in TNBC are not yet comprehensively elucidated.

A notable aspect of cancer progression is the metabolic reprogramming of both tumor cells and immune cells within the tumor microenvironment [16]. CD8 + T cells are a crucial component of the antitumor immune response, and their functionality is closely tied to their metabolic state [17]. Glucose metabolism, particularly glycolysis, has been recognized as a pivotal regulator of CD8 + T cell activation and effector functions [18].

The activation of T-cells, which is vital for mounting an effective antitumor immune response, depends on the PI3K/AKT/mTOR signaling pathway [19]. Indeed, AKT boosts glucose metabolism by elevating the expression of glucose transporter 1 (GLUT1), consequently promoting glucose uptake in T-cells [20]. As a result, mTOR signaling serves as the integrator of immune signals and metabolic cues within T-cells [21].

In light of these considerations, investigating the interplay between the CD155/TIGIT pathway and the metabolic behavior of CD8 + T cells in the context of TNBC holds promise for enhancing our understanding of tumor immune evasion and identifying potential therapeutic targets. The objective of this study is to uncover the mechanistic connections between CD155/TIGIT signaling, the glucose metabolism of CD8 + T cells, and the progression of TNBC. This research aims to provide valuable insights that could lead to the formulation of innovative approaches for managing this particularly aggressive form of breast cancer.

## Materials and methods

### Patients and tissue samples

We obtained a total of 20 pairs of TNBC tumor tissues and adjacent normal tissues from Fujian Medical University Union Hospital. Ethical approval for this study was granted by the ethics committee of Fujian Medical University Union Hospital.

### Cell isolation

We employed Ficoll (Cytiva, USA) density gradient centrifugation to isolate PBMCs from whole blood. Single-cell suspensions were prepared from fresh tumor tissue using a combination of mechanical dissociation, following the manufacturer's guidelines with a gentle MACS C tube (Milteny Biotec, Bergisch Gladbach, Germany), and enzymatic hydrolysis using a tumor dissociation kit (Milteny Biotec). Following the digestion process, the cells were filtered through a 70 µm mesh, subjected to centrifugation with Ficoll (Cytiva, USA), and subsequently, monocytes were resuspended in RPMI-1640.

### Cell culture

The 4T1 and MDA-MB-231 cell lines were procured from the Academy of Medical Sciences (Beijing, China). These cell lines were cultured in DMEM supplemented with 10% fetal bovine serum (Gibco, Grand Island, NY, USA), along with 50 U/mL penicillin and 50 mg/mL streptomycin. CD8 + T cells were isolated from PBMCs using positive selection with a kit from Miltenyi Biotec (Bergisch Gladbach, Germany). Subsequently, these CD8 + T cells were activated utilizing an anti-CD3/CD28 antibody also from Miltenyi Biotec (Bergisch Gladbach, Germany). Following activation, CD8 + T cells were co-cultured with TNBC cells in 48-well plates at a 10:1 ratio. Subsequently, a concentration of 5 μg/ml of anti-TIGIT mAb Tiragolumab (Selleckchem, USA) was added to the cell culture. As a control, α-human IgG1 (Selleckchem, USA) was used as an isotype control. Finally, CD8 + T cells were isolated and purified with magnetic beads from the co-culture system for subsequent experiments.

### Flow cytometry

PBMCs, isolated from a healthy volunteer, were labeled with FITC-conjugated anti-CD8 antibodies (BD Bioscience, USA) and incubated for 30 min at 4 °C. To perform intracellular staining, the cells, which had been previously labeled with antibodies against cell surface markers, underwent fixation and permeabilization using a Transcription Factor Buffer Set (BD Bioscience, USA) for a duration of 20 min to destroy the cell membrane and nuclear membrane. Afterward, they were treated with fluorochrome-conjugated antibodies, including APC-conjugated anti-TNFα (BD Pharmingen, USA), BV421-conjugated anti-IFNγ (BD Bioscience, USA), PE-conjugated anti-Ki67 (BD Bioscience, USA), and PE-conjugated anti-Granzyme B (BD Bioscience, USA). The intracellular staining was conducted at 4 °C for a duration of 30 min. In the end, the analyzed cells were run through a FACS Calibur flow cytometer provided (Becton Dickinson, USA), and the resulting data were processed using FlowJo software.

### Immunohistochemistry (IHC)

The sections underwent initial deparaffinization, followed by heat-mediated antigen retrieval using citric acid buffer. Following the provided manufacturer's guidelines, an immunohistochemistry detection kit from Zhongshan Jinqiao (Beijing, China) was employed for the assay. The sections were incubated overnight at 4 °C with primary antibodies in PBS, including anti-human TIGIT (1:100, Cell Signaling Technology) and anti-human CD155 (1:100, Cell Signaling Technology, Danvers). Following that, the sections underwent a 10-min incubation at 37 °C with a biotin-labeled secondary antibody (goat anti-rabbit IgG). Subsequently, sections were treated with streptavidin-conjugated peroxidase for 15 min at 37 °C, and staining was accomplished using DAB (Zhongshan Jinqiao, Beijing, China). Hematoxylin was employed for a 5-min section staining. Following dehydration, the sections were sealed with neutral resin.

### Multiplex immunohistochemistry (mIHC)

In our mIHC analysis, we utilized specialized kits for multiple fluorescence immunohistochemical staining from Absin (Shanghai, China). The steps for heat-mediated antigen retrieval and primary antibody incubation were carried out following the identical procedures outlined for immunohistochemistry. Subsequently, following a 10-min incubation with the secondary antibody, the sections underwent further incubation with the fluorescent staining amplification solution for an additional 10 min at 37 °C. After being subjected to three washes with TBST, the sections were treated with 4',6-diamidino-2-phenylindole (DAPI) for a duration of 5 min. To conclude, an anti-fluorescence quenching agent was applied to seal the slides.

### Western blot

First, we purified CD8 + T cells from a co-culture system with TNBC cells using magnetic beads. After undergoing three washes with PBS, the CD8 + T cells were subsequently lysed on ice with radioimmunoprecipitation analysis buffer (RIPA; Beyotime, China Institute of Biotechnology), which was supplemented with 1% phenylmethylsulfonyl fluoride (PMSF) and 1% NaF, and this lysis process lasted for 30 min. Subsequently, the samples were centrifuged at 12,000 rpm for 10 min at 4 °C to collect the supernatant. Following this step, the proteins were separated on SDS-PAGE gels and then transferred to a PVDF membrane (Merck Millipore, Burlington, Massachusetts, USA). The PVDF membrane was then subjected to incubation with primary antibodies targeting the following proteins: GAPDH (1:1000, Cell Signaling Technology), GLUT1 (1:1000, Cell Signaling Technology), HK2 (1:1000, Cell Signaling Technology), PKM2 (1:1000, Cell Signaling Technology), LDHA (1:1000, Cell Signaling Technology), PI3K (1:1000, Cell Signaling Technology), AKT (1:1000, Cell Signaling Technology), p-AKT (1:1000, Cell Signaling Technology), mTOR (1:1000, Cell Signaling Technology), and p-mTOR (1:1000, Cell Signaling Technology). The overnight incubation took place at 4 °C. Following that, the membrane was subjected to incubation with the corresponding secondary antibody. Image J software, provided by the National Institutes of Health, was utilized to assess relative protein levels, employing GAPDH as the internal control.

### Glucose consumption assay

Glucose concentrations were analyzed in triplicate using a Glucose Assay Kit (Abcam, USA) according to the manufacturer's instructions.

### Lactate production assay

Lactate concentrations were analyzed in triplicate using a Lactate Assay Kit (Abcam, USA) according to the manufacturer's instructions.

### In vivo treatments

For this study, we acquired female BALB/c mice aged 4 to 6 weeks from Beijing Vital River Laboratory Animal Technology Co., Ltd. The 4T1 cells were prepared by trypsinization, suspended in PBS, and then each mouse received a subcutaneous injection of 200 ml of this cell suspension, equivalent to 1 × 10^7^ cells. To conduct in vivo blockade experiments, 7 days post cell suspension injection, the mice were randomly divided into two groups. One group received anti-TIGIT mAb (100 μg, clone 1G9, BioXcell, West Lebanon, USA), while the other group received IgG (mouse IgG1, clone MOPC-21, BioXcell). Intraperitoneal injections of the blocking antibody and the isotype control were administered on days 5, 11, 14, and 17. Furthermore, the mice were subcutaneously inoculated with either 1 × 10^7^ 4T1-NC-CD155 or 4T1-KO-CD155 cells to investigate the therapeutic effects of targeted CD8 + T cells against tumors. We extracted lymphocytes from tumor tissues for further flow analysis. Care of animals was in accordance with institution guidelines.

### Bioinformatics analysis

Gene expression data and clinical annotations were sourced from GEO (Gene Expression Omnibus) and TCGA (The Cancer Genome Atlas). To analyze differentially expressed genes between TNBC and normal tissues, the "limma" package was employed. Furthermore, CD8 + T cell infiltration levels were assessed using algorithms like MCPCOUNTER, XCELL, and QUANTISEQ.

### Statistical analysis

Statistical significance was determined using GraphPad Prism 9.0 software (GraphPad Software, San Diego, CA), and the findings are presented as the mean ± standard deviation. To compare two independent groups, the two-tailed Student’s t-test was employed, while a nonparametric test was used when the data did not conform to a normal distribution. A p-value less than 0.05 across all experiments was regarded as indicative of statistical significance, and the significance levels are denoted as follows: *P < 0.05, **P < 0.01.

## Results

### CD155 was highly expressed in TNBC and associated with down-regulation of CD8^+^T cells infiltration

**Fig. 1:**
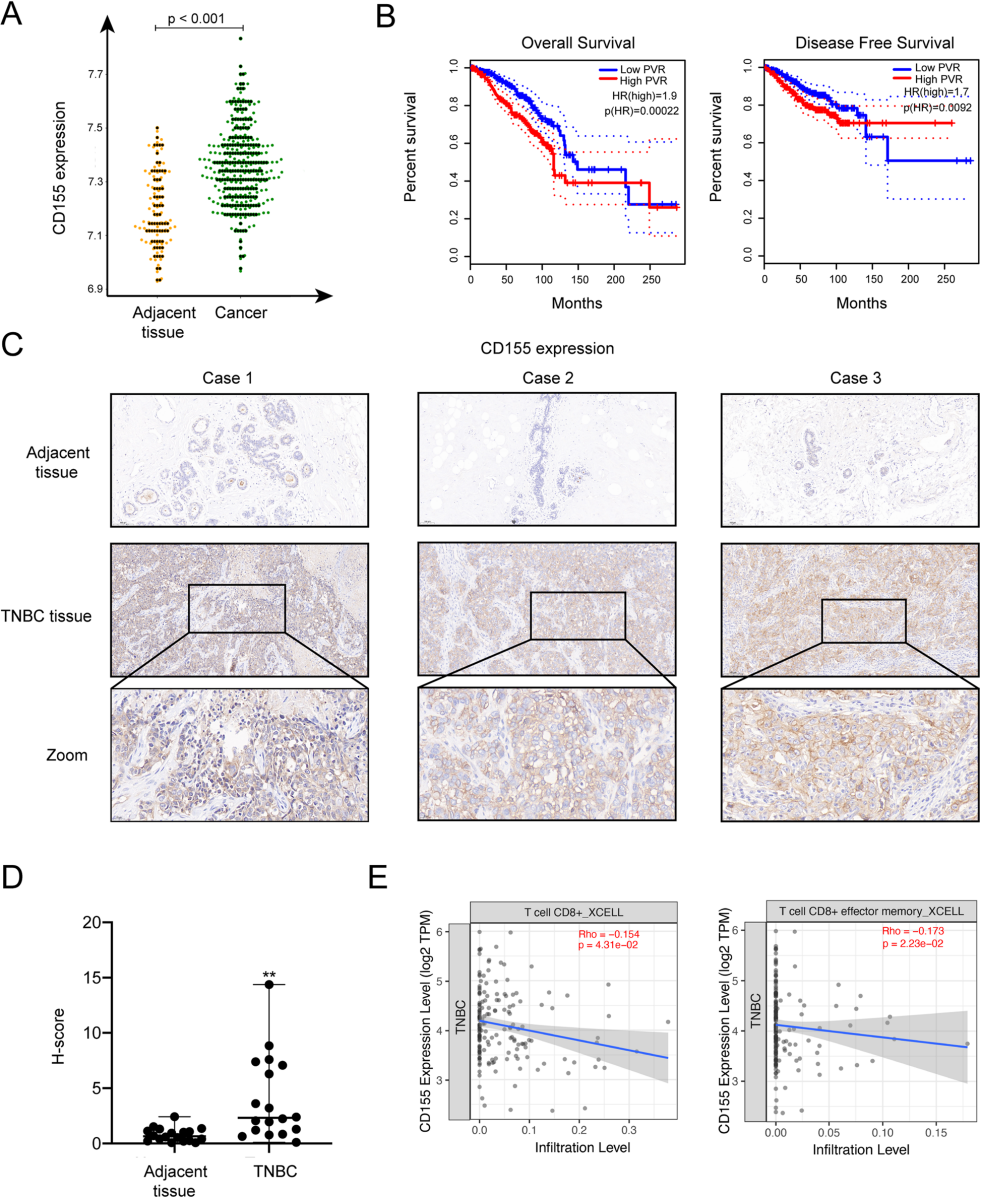
CD155 is highly expressed in TNBC and was associated with down-regulation of CD8.^+^T cells infiltration. **A** The analysis of CD155 expression in both cancerous and adjacent tissues of TNBC patients was conducted using the GEO database (GSE76275); **B** We examined the relationship between CD155 expression and the overall survival (OS) and disease-free survival (DFS) of TNBC patients using data from the GEO database (GSE76275); **C** The expression of CD155 in cancer and adjacent tissues was analyzed by IHC (*n* = 20); **D** Quantitative immunohistochemical assessment of CD155 expression in cancer and adjacent tissues (*n* = 20); **E** We conducted a correlation analysis, assessing the relationship between CD155 expression levels and the degree of CD8 + T cell infiltration within TCGA datasets, utilizing the XCELL algorithm. The data are the mean ± SEM of the experiments. *P < 0.05, **P < 0.01

We conducted an in-depth analysis of the differential expression of CD155 within TNBC and adjacent tissues, utilizing data sourced from GEO databases (GSE76275). Our primary objective was to elucidate the potential involvement of CD155 in the progression of TNBC. CD155 exhibited significantly elevated expression levels in individuals with TNBC compared to those without the condition (Fig. 1A). Moreover, elevated CD155 expression exhibited a significant association with reduced overall survival (OS) and disease-free survival (DFS), implying an unfavorable correlation between CD155 levels and the prognosis of TNBC patients (Fig. 1B). To further validate the aforementioned results, we collected tissue samples from 20 TNBC patients and performed IHC analysis to assess the expression of CD155 in both cancerous and adjacent tissues. The findings indicated a significantly elevated expression level of CD155 in cancerous tissues compared to adjacent tissues (Fig. 1C and D). A substantial body of research has indicated that CD155 participates in the immune evasion mechanisms of a wide range of cancers, primarily by modulating the activation and infiltration of CD8^+^T cells within the tumor microenvironment [22, 23]. Hence, we employed XCELL to examine the relationship between CD155 and CD8 + T cell infiltration in TNBC, and observed that CD155 expression in TNBC patients was associated with a reduction in CD8 + T cell infiltration (Fig. 1E).

### TIGIT was highly expressed in patients with TNBC

**Fig. 2:**
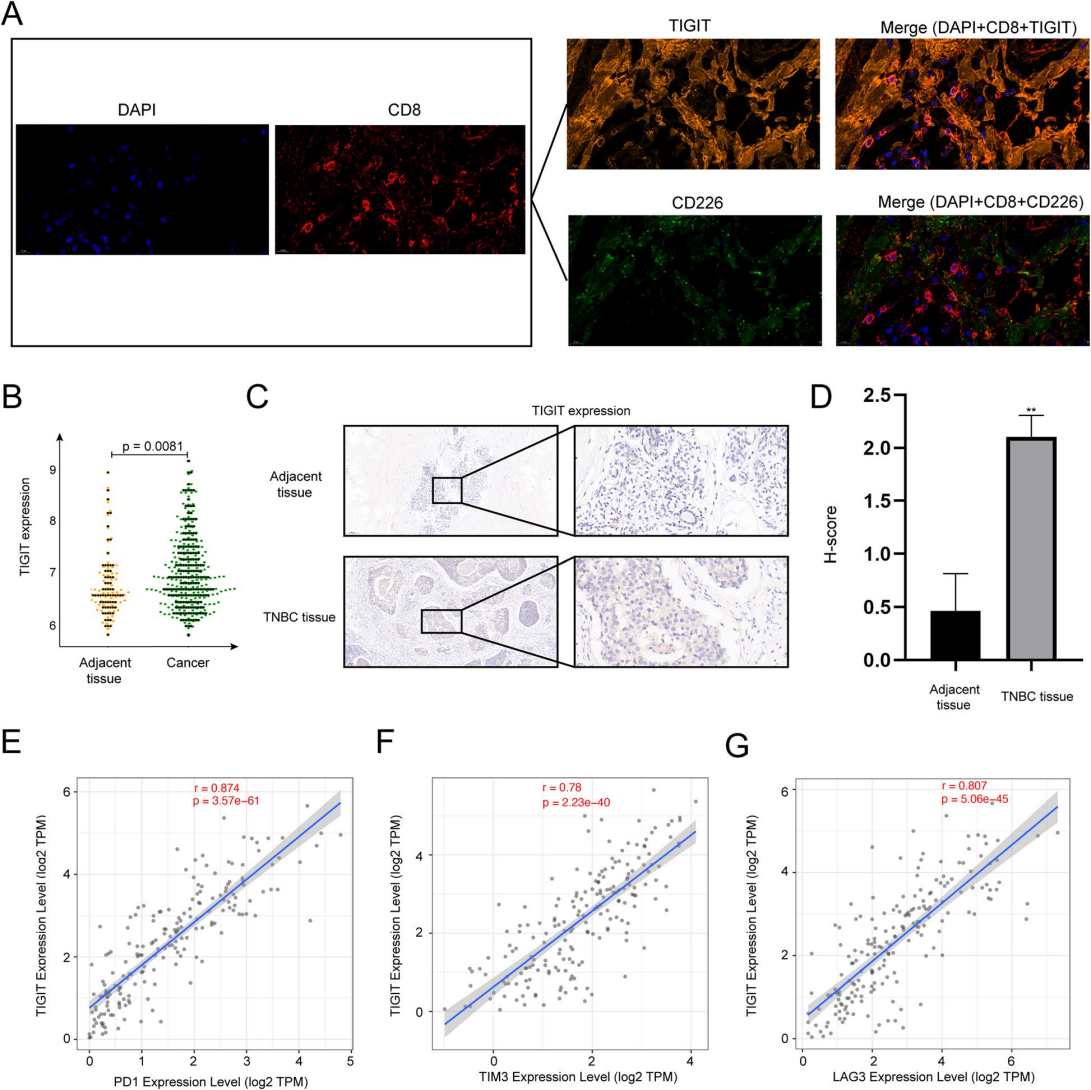
TIGIT was highly expressed in TNBC. **A** We employed mIHC to analyze the expression of TIGIT and CD226 on CD8 + T cells in TNBC tissues; **B** The expression of TIGIT in cancer and adjacent tissues was evaluated using the GEO database; **C** TIGIT expression in both cancerous and adjacent tissues was assessed through IHC, with a sample size of *n* = 3; **D** TIGIT expression was quantitatively evaluated through immunohistochemistry in both cancerous and adjacent tissues, with a sample size of *n* = 3; **E** The TCGA database was utilized to investigate the correlation between TIGIT expression and PD1 expression in TNBC tissues; **F **The TCGA database was employed to examine the correlation between TIGIT expression and TIM3 expression in TNBC tissue; **G** The TCGA database was utilized to analyze the correlation between TIGIT expression and LAG3 expression in TNBC tissues. The data are the mean ± SEM of the experiments

CD155 is a high-affinity ligand for both TIGIT and CD226 [7]. However, TIGIT can effectively inhibit the interaction between CD155 and CD226 [24], providing further evidence of TIGIT's superior affinity for CD155. We performed mIHC to analyze the expression of TIGIT and CD226 on CD8 + T cells. The results showed that the expression level of TIGIT on CD8^+^T cells in TNBC was higher than that of CD226 (Fig. 2A), suggesting that CD155 in TNBC tended to bind to TIGIT. To investigate the potential role of TIGIT in TNBC progression, we initially utilized the GEO database to analyze the expression of TIGIT in both cancer and C tissues of TNBC patients and the results indicated a notable increase in TIGIT expression within cancerous tissues compared to adjacent normal tissues (Fig. 2B). Notably, the IHC results obtained from both cancer and adjacent tissues also confirmed the significantly elevated expression of TIGIT in TNBC (Fig. 2C and D). The co-expression of immune checkpoint molecules has the potential to enhance immunosuppression [14, 25]. Consequently, we examined the expression of TIGIT alongside co-inhibitory receptors PD-1, T cell immunoglobulin and mucin domain-containing protein 3 (Tim3) and lymphocyte activation gene 3 protein (LAG3) in TNBC. Our observations revealed a positive correlation between TIGIT and PD-1, Tim3 and LAG3 in TNBC patients (Fig. 2 E, F and G). Since PD-1, Tim3 and LAG3 can inhibit the immune activation of CD8^+^T cells, it is reasonable to speculate that TIGIT can inhibit the immune activation of CD8^+^T cells in TNBC.

### TIGIT blockade reverse the inhibitory effect of TNBC on CD8^+^T cells

**Fig. 3:**
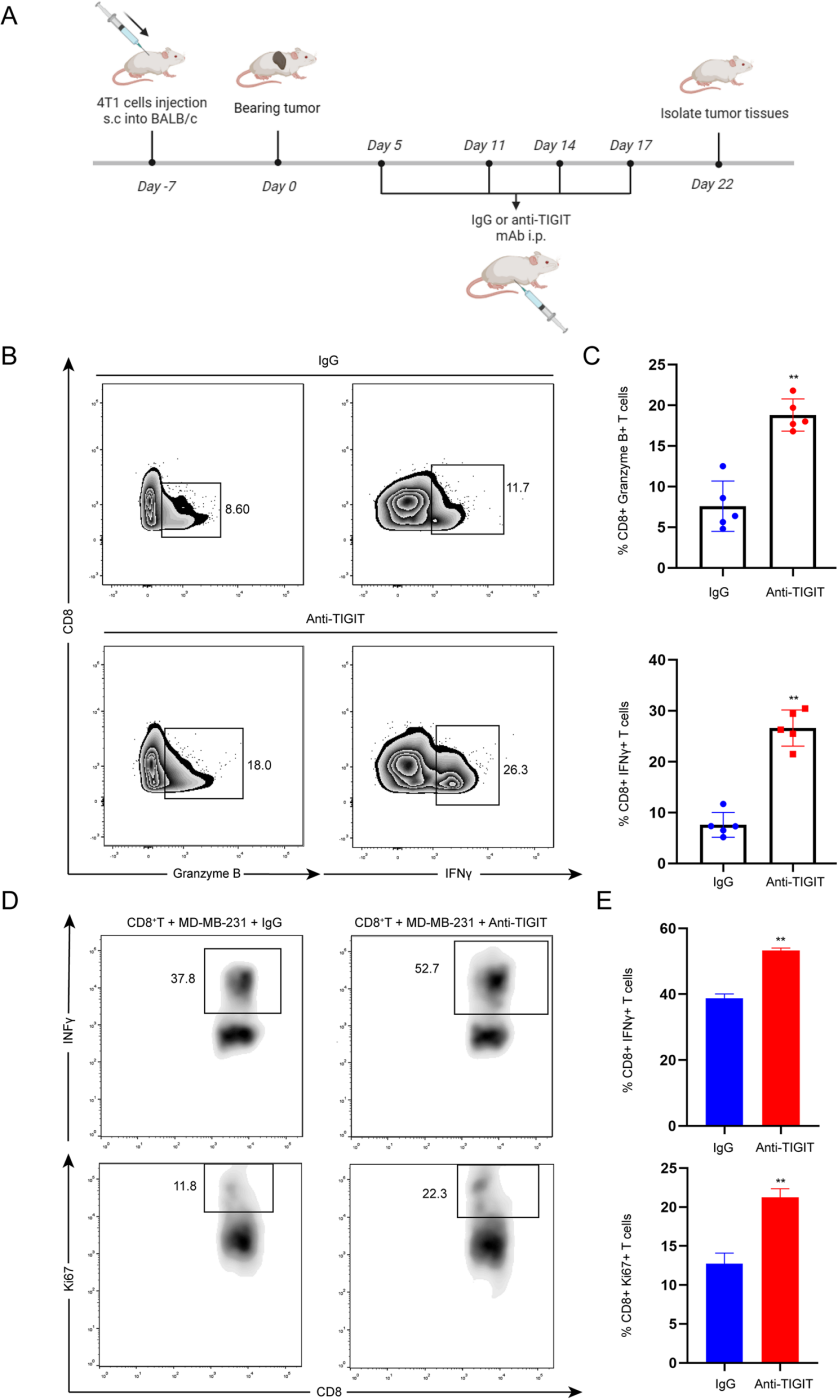
TIGIT blockade reverse the inhibitory effect of TNBC on CD8 + T cells. **A** Experimental design for in vivo mouse experiments; **B** Flow cytometry was used to measure the levels of Granzyme B and IFNγ secreted by CD8 + T cells from tumor tissues in mice receiving different treatments (*n* = 5); **C** Percentage of CD8 + T cells from tumor tissues producing Granzyme B and IFNγ; **D** In an in vitro setting, CD8 + T cells extracted from human PBMC were co-cultured with TNBC cells in the presence of either an anti-TIGIT antibody or an isotype control. Flow cytometry was employed to measure the production of IFNγ and Ki67 in CD8 + T cells; **E** Percentage of CD8 + T cells producing IFNγ and Ki67. The data are the mean ± SEM of the experiments

The aforementioned findings indicate the potential involvement of the CD155/TIGIT signaling pathway in suppressing the immune activation of CD8 + T cells in TNBC. To further validate these results, we proceeded to establish an in vivo mouse subcutaneous tumor-bearing model, where mice received intraperitoneal injections of either IgG or Anti-TIGIT (Fig. 3A). Our results demonstrated the reversal of Granzyme B and IFNγ inhibition by CD8 + T cells from tumor tissues following TIGIT blockade (Fig. 3B and C). Additionally, we conducted in vitro co-cultures of CD8 + T cells extracted from human PBMC with TNBC cells in the presence of an anti-TIGIT blocking antibody or isotype control. The findings indicated that TIGIT blocking led to the restoration of IFNγ and Ki-67 secretion by CD8 + T cells (Fig. 3D and E). In summary, TIGIT blockade reversed the inhibitory effect of TNBC on CD8 + T cells.

### CD155/TIGIT blockade reverse the inhibitory effect of TNBC on CD8 + T cells

**Fig. 4:**
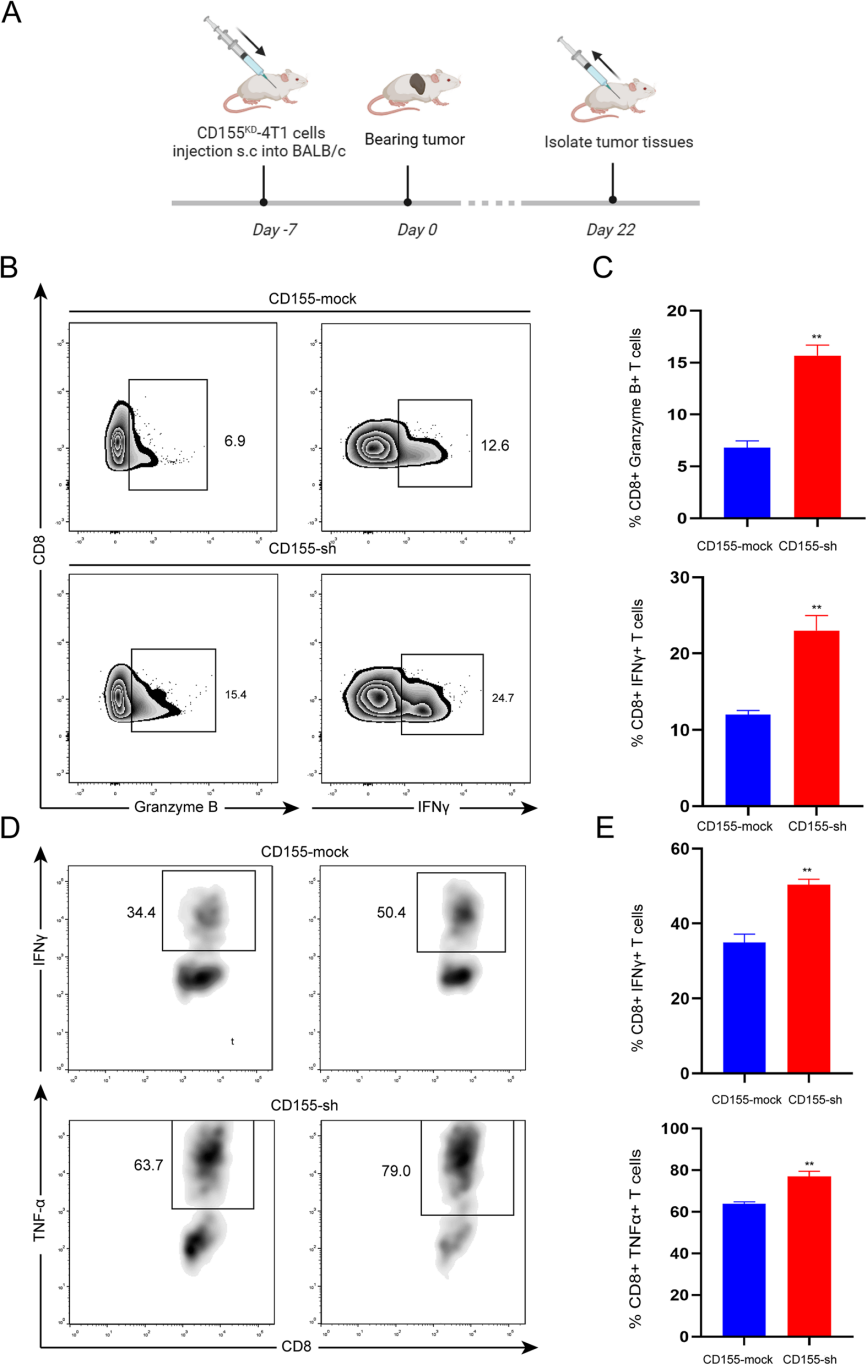
CD155/TIGIT blockade reverse the inhibitory effect of TNBC on CD8 + T cells. **A** Experimental design of mice in vivo; **B** The levels of Granzyme B and IFNγ secreted by CD8 + T cells from tumor tissues in tumor bearing model constructed by CD155^KD^ or CD155^mock^ TNBC cells were measured by flow cytometry (*n* = 5); **C** Percentages of Granzyme B and IFNγ-producing CD8 + T cells from tumor tissues; **D** CD8 + T cells were cocultured with CD155^KD^ or CD155^mock^ TNBC cells. The production of IFNγ and TNFα in CD8 + T cells were measured by flow cytometry; **E** Percentages of IFNγ and TNFα-producing CD8 + T cells. The data are the mean ± SEM of the experiments

To further substantiate that TIGIT exerts immunosuppressive effects in TNBC via the CD155 ligand, we established a TNBC cell line with CD155 knockdown and examined its impact on CD8 + T cells both in vivo (Fig. 4A) and in vitro. Our findings revealed that the inhibition of Granzyme B and IFNγ by CD8 + T cells from tumor tissues was reversed upon CD155 knockdown (Fig. 4B and C). Additionally, we co-cultured CD8 + T cells with CD155-knockdown TNBC cells in vitro, and the results indicated that CD155 knockdown led to the restoration of IFNγ and TNFα secretion by CD8 + T cells (Fig. 4D and E). Get together, CD155/TIGIT blockade reverse the inhibitory effect of TNBC on CD8 + T cells.

### CD155/TIGIT signaling mediates glycose metabolism inhibition of CD8 + T cells by TNBC cells

**Fig. 5:**
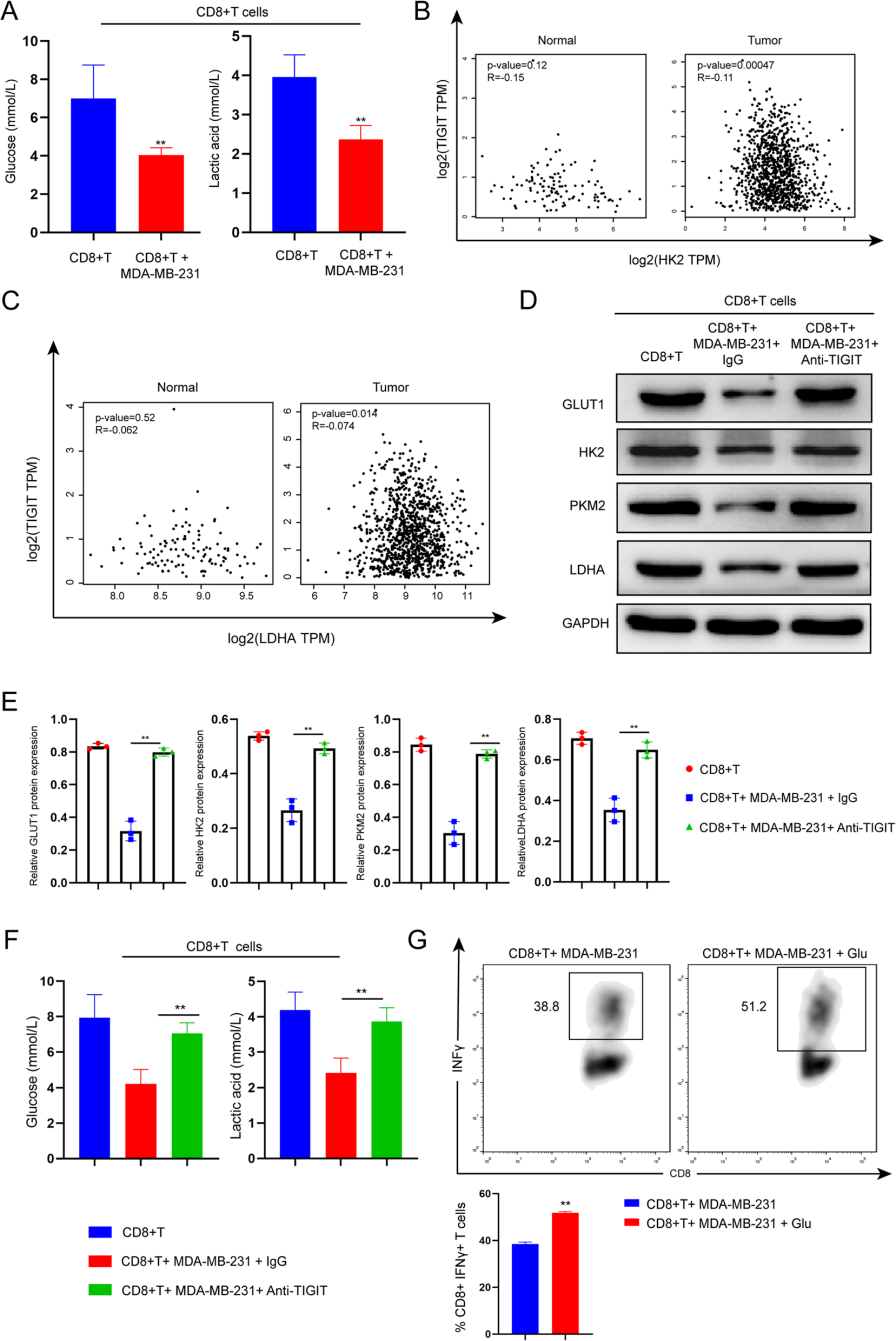
CD155/TIGIT signaling mediates glycose metabolism inhibition of CD8 + T cells by TNBC cells. **A** Glucose and lactic acid level of CD8 + T cells with or without cocultured with TNBC cells; **B** GEPIA analyzed the correlation between TIGIT and HK2 expression in breast cancer and normal tissues; **C** GEPIA analyzed the correlation between TIGIT and LDHA expression in breast cancer and normal tissues; **D** Activated CD8 + T cells were cocultured with TNBC cells in the presence of an anti-TIGIT blocking antibody. Western blot analysis of GLUT1, HK2, PKM2 and LDHA expression in CD8 + T cells in different groups; **E** Relative protein expression of GLUT1, HK2, PKM2 and LDHA; **F** Glucose and lactate levels of CD8 + T cells co-cultured with TNBC cells in the presence or absence of anti-TIGIT blocking antibody; **G** CD8 + T cells co-cultured with TNBC cells were treated with or without glucose. IFNγ production was measured by flow cytometry. The data are the mean ± SEM of the experiments

Glucose uptake and glycolysis increase rapidly when T cells are activated [26]. Alterations in the glycolytic metabolism of CD8 + T cells play a central role in shaping their activation and functionality. It's worth highlighting that glycolytic processes are highly relevant both in the functional decline and the subsequent restoration of CD8 + T cells [27]. To investigate the impact of TNBC on the glucose metabolism of CD8 + T cells, we assessed the levels of glucose and lactic acid in CD8 + T cells following co-culture with TNBC cells. Our findings demonstrated a significant reduction in both glucose and lactic acid levels in CD8 + T cells after co-culture with TNBC cells (Fig. 5A). GLUT1 has been recognized as a crucial player in glucose uptake by T cells [14]; Hexokinase 2 (HK2) and Pyruvate kinase isoform M2 (PKM2) represent pivotal enzymes within the glycolysis pathway, while Lactate dehydrogenase A (LDHA) is responsible for catalyzing the conversion of pyruvate into lactic acid during glycolysis [28]. The results of the GEPIA analysis revealed a correlation between TIGIT expression and the down-regulation of HK2 and LDHA in breast cancer tissues. However, this correlation was not observed in normal tissues, indicating that TIGIT may exert an inhibitory effect on glycolytic pathways specifically in the context of breast cancer (Fig. 5B and C). Subsequent western blot results indicated that co-culturing with MDA-MB-231 cells led to the suppression of GLUT1, HK2, PKM2, and LDHA expression in CD8 + T cells. However, TIGIT blocking effectively reversed the inhibitory effects of TNBC cells on the expression of glycolytic enzymes in CD8 + T cells (Fig. 5D and E). Additionally, TIGIT blocking also reversed the inhibition of glucose and lactic acid levels in CD8 + T cells induced by MDA-MB-231 cells (Fig. 5F). Crucially, flow cytometry results highlighted that glucose treatment effectively counteracted the inhibitory effect of MDA-MB-231 cells on the secretion of IFNγ by CD8 + T cells (Fig. 5G). In summary, these findings suggest that TNBC cells impede the glycolysis of CD8 + T cells through the CD155/TIGIT signaling pathway.

### CD155/TIGIT signaling disrupts CD8 + T cell glycose metabolism by inhibiting activation of the PI3K/AKT/mTOR pathway

**Fig. 6:**
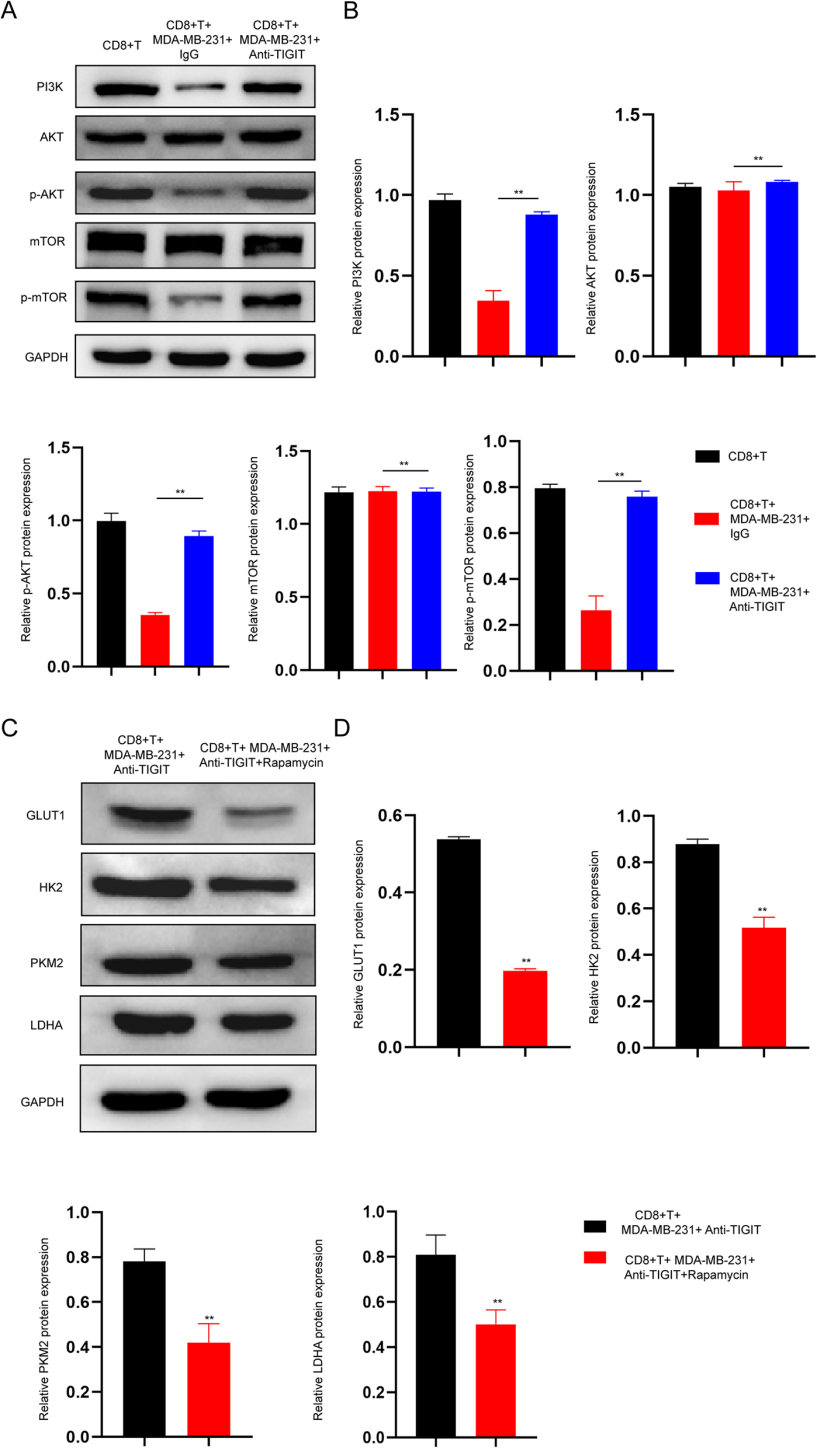
CD155/TIGIT signaling disrupts CD8 + T cell glycose metabolism by inhibiting activation of the PI3K/AKT/mTOR pathway. **A** CD8 + T cells co-cultured with TNBC cells in the presence or absence of anti-TIGIT blocking antibody. Western blot analysis of PI3K, AKT, p-AKT, mTOR and p-mTOR expression in CD8 + T cells in different groups; **B** Relative protein expression of PI3K, AKT, p-AKT, mTOR and p-mTOR; **C** Activated CD8 + T cells treated with rapamycin during co-culture with TNBC cells in the presence of anti-TIGIT blocking antibody. Western blot analysis of GLUT1, HK2, PKM2 and LDHA expression in CD8 + T cells; **D** Relative protein expression of GLUT1, HK2, PKM2 and LDHA

PI3K/AKT/mTOR is a major signaling pathway that activates the glycolytic pathway [29]. Next, we analysed the changes in related proteins in CD8 + T cells co-cultured with TNBC cells in the presence or absence of anti-TIGIT blocking antibody. Co-culture with TNBC cells significantly decreased the expression of PI3K, p-AKT and p-mTOR, while blocking TIGIT reversed the inhibition of TNBC on the expression of these three proteins in CD8 + T cells (Fig. 6A and B). Rapamycin is a potent and specific inhibitor of mTOR [30]. Notably, activated CD8 + T cells treated with rapamycin during co-culture with TNBC cells in the presence of anti-TIGIT blocking antibody reduced the expression of glycolytic-related enzymes (GLUT1, HK2, PKM2 and LDHA) (Fig. 6C and D). In summary, CD155/TIGIT signaling disrupts CD8 + T cell glycose metabolism by inhibiting activation of the PI3K/AKT/mTOR pathway.

### Targeting CD155/TIGIT to inhibit tumor progression in vivo

**Fig. 7:**
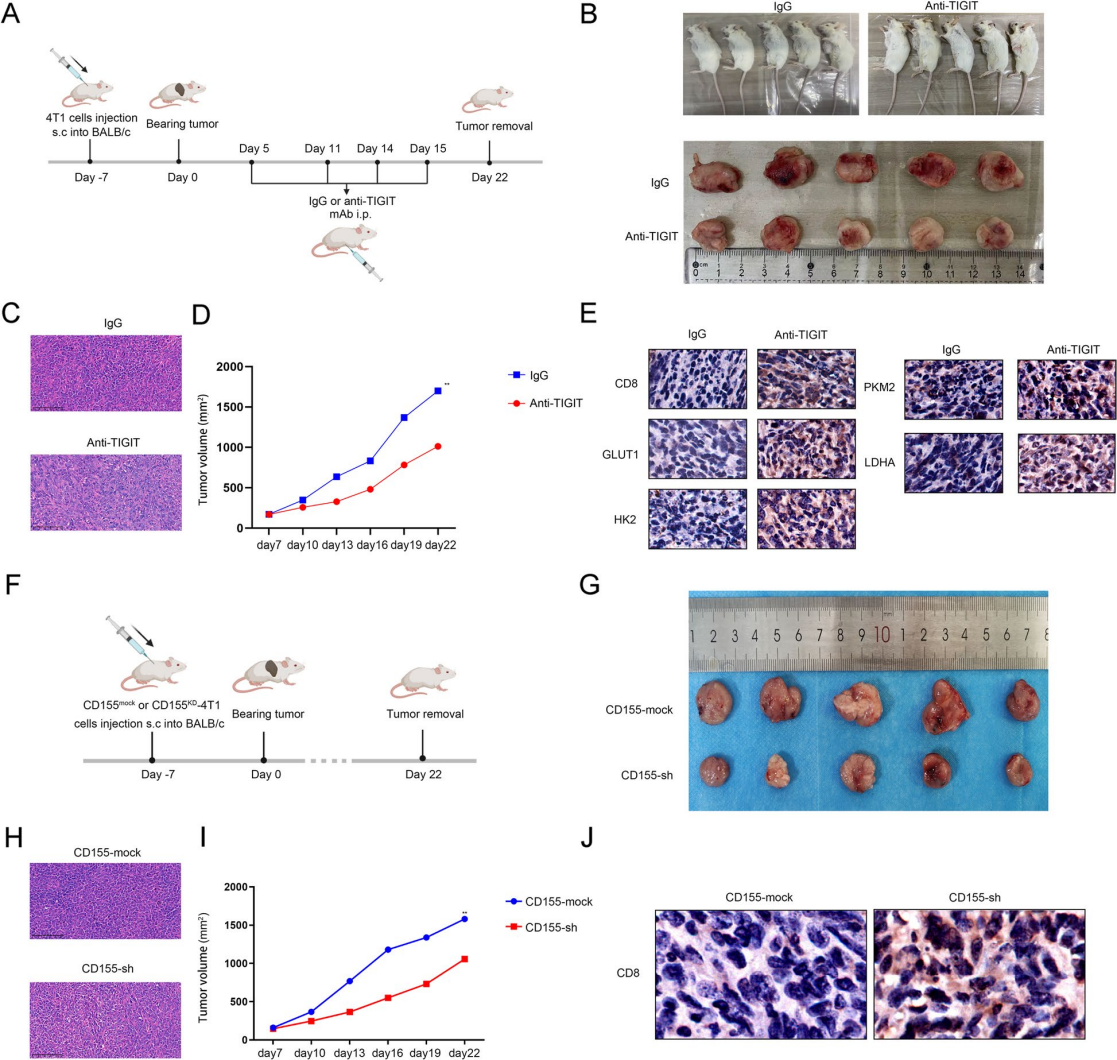
Targeting CD155/TIGIT to inhibit tumor progression in vivo. **A **Tumor bearing model by subcutaneously injecting 4T1 cells into BALB/c mice, and treated them with anti-TIGIT mAb or IgG; **B** Tumor size in mice treated with anti-TIGIT mAb or IgG; **C** HE staining of mouse tumor tissue treated them with anti-TIGIT mAb or IgG; **D** Tumor volume in mice that were treated them with anti-TIGIT mAb or IgG; **E** IHC analyzed the expression of CD8, GLUT1, HK2, PKM2 and LDHA in tumor tissues treated them with anti-TIGIT mAb or IgG; **F** Tumor bearing model by subcutaneously injecting 4T1-CD155^KD^ or 4T1-CD155^mock^ cells into BALB/c mice; **G** Tumor size in mice treated with anti-TIGIT mAb or IgG; **H** HE staining of mouse tumor tissues that were injected with 4T1-CD155^KD^ or 4T1-CD155^mock^ cells; **I** Tumor volume in mice that were injected with 4T1-CD155^KD^ or 4T1-CD155^mock^ cells; **J** IHC analysis of CD8 expression in tumor tissue injected with 4T1-CD155^KD^ or 4T1-CD155^mock^ cells

We first constructed a tumor bearing model by subcutaneously injecting 4T1 cells into BALB/c mice, and treated them with anti-TIGIT mAb or IgG (Fig. 7A). The extracted tumor tissue was confirmed as TNBC by HE staining (Fig. 7C), and it was found that tumor progression was significantly inhibited in mice treated with anti-TIGIT mAb (Fig. 7B and D). In addition, the results of IHC analysis showed that the expressions of CD8, GLUT1, HK2, PKM2 and LDHA were significantly up-regulated in tumor tissues treated with anti-TIGIT mAb (Fig. 7E). It was suggested that TIGIT blockade activates glycolytic pathway in CD8 + T cells to enhance anti-TNBC immunity. Moreover, we established a tumor-bearing model model by subcutaneously injecting 4T1-CD155^mock^ or 4T1-CD155^KD^ cells to verify the antitumor efects of targeting TIGIT/CD155 signalling in vivo (Fig. 7F). The extracted tumor tissue was confirmed as TNBC by HE staining (Fig. 7H), and tumor progression was inhibited in mice that received 4T1-CD155^KD^ cells (Fig. 7G and I). Taken together, targeting the CD155/TIGIT signaling pathway inhibits TNBC progression in vivo.

## Discussion

Immunotherapy has now taken its place alongside surgery, radiotherapy, chemotherapy, and targeted therapy, establishing itself as a significant approach to cancer treatment [31]. Immune escape is a dysfunction of the immune system that facilitates the development of cancer. It is driven by the increased expression of immune checkpoints, such as PD-1, leading to T-cell exhaustion [32]. CD8 + T cells play a crucial role as the primary effector cells in antitumor immunity. However, in tumor-bearing hosts, these cells become exhausted and lose their functionality due to the presence of immune checkpoints [33]. Research into the immunosuppressive impact of TIGIT on CD8 + T cells is growing. However, the role and mechanism of CD155/TIGIT in TNBC have not yet been thoroughly investigated. An analysis of GEO databases showed higher CD155 expression in TNBC tumor tissue than that in normal tissue, and it was related to the shorter survival of patients with TNBC. The results of IHC also revealed that the expression level of CD155 in cancerous tissues was significantly higher than in adjacent tissues. Our study demonstrated that TIGIT blockade reverse the inhibitory effect of TNBC on CD8 + T cells. Targeting CD155/TIGIT pathway suppressed tumor progression and improved survival in tumor bearing mice.

Activated T cells rely on sufficient energy resources and undergo alterations in cellular metabolism to mount effective antitumor immune responses [34]. Our findings revealed a compelling connection between CD155/TIGIT signaling and the modulation of CD8 + T cell glucose metabolism. The interaction between CD155 on tumor cells and TIGIT on CD8 + T cells appeared to disrupt the balance of glucose utilization, affecting the metabolic fitness of CD8 + T cells. This disruption likely contributes to the impairment of CD8 + T cell activation and effector functions observed in the tumor microenvironment. Importantly, this study sheds light on a novel mechanism through which TNBC cells exploit the CD155/TIGIT axis to rewire CD8 + T cell metabolism, thus creating an immunosuppressive milieu that supports tumor progression. The implications of these findings extend beyond metabolic regulation. The CD155/TIGIT axis, known for its immunosuppressive effects, appears to orchestrate a multi-dimensional strategy for promoting TNBC progression. By impairing CD8 + T cell glucose metabolism, the tumor gains the ability to not only evade immune surveillance but also to establish an environment that may support its growth and spread. This study underscores the intricate crosstalk between immune checkpoints and cellular metabolism, providing a foundation for potential therapeutic interventions aimed at restoring CD8 + T cell functionality and countering TNBC progression.

Additionally, in the current study, we found that TNBC cells deprived CD8 + T cells of glucose and downregulated the PI3K/AKT/mTOR metabolic pathway in CD8 + T cells. These findings suggest that TNBC cells inhibit PI3K/AKT/mTOR signaling pathway in CD8 + T cells, which resulted in reduced glucose uptake and lactate production. Furthermore, our research revealed that blocking TIGIT led to the activation of metabolic pathways in CD8 + T cells. Specifically, TIGIT blockade increased the activation of the PI3K/AKT/mTOR pathway, subsequently enhancing glucose metabolism and cytokine production in CD8 + T cells. These results highlight the role of TIGIT signaling in suppressing the PI3K/AKT/mTOR pathway, thereby inhibiting glucose metabolism in CD8 + T cells. PD-1 has been shown to inhibit the activation of the mTOR pathway and suppress glycolysis in T cells [35]. These immune checkpoints may share some common mechanisms in regulating T-cell function. However, it's important to note that the signaling mechanism is distinct from a previous report suggesting that TIGIT signals through ZAP70 and ERK1/2 in NK cells [36]. In summary, our findings indicate that gastric cancer cells inhibit the PI3K/AKT/mTOR pathway in CD8 + T cells by upregulating TIGIT expression on CD8 + T cells. These results prompt a broader consideration of therapeutic strategies for TNBC. Combination therapies targeting both the CD155/TIGIT axis and metabolic pathways could potentially enhance the efficacy of immunotherapies and metabolic interventions. Developing such strategies requires a comprehensive understanding of the intricate molecular mechanisms underlying these processes, emphasizing the need for further investigations into the signaling cascades and cellular interactions involved.

**Fig. 8:**
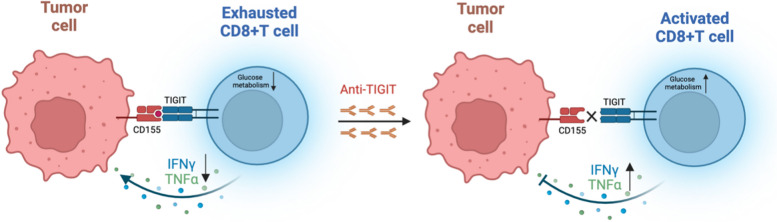
Schematic diagram of CD155/TIGIT binding to regulate CD8 + T cell function and related mechanisms

In conclusion, this study uncovers a previously unrecognized mechanism by which CD155/TIGIT signaling promotes TNBC progression through the modulation of CD8 + T cell glucose metabolism via inhibited PI3K/AKT/mTOR pathway (Fig. 8). By disrupting the metabolic equilibrium of immune cells, TNBC cells manipulate the immune microenvironment to their advantage. These findings pave the way for novel therapeutic avenues that simultaneously target immunosuppression and metabolic reprogramming, potentially offering a more effective strategy for combatting TNBC.

### Supplementary Information

**Additional file 1. **Full uncropped gels and blots.

## Data Availability

The data used to support the findings of this study are available from the corresponding author upon request.
