# Supplementary material for: The immune checkpoint TIGIT/CD155 promotes the exhaustion of CD8 + T cells in TNBC through glucose metabolic reprogramming mediated by PI3K/AKT/mTOR signaling
Source: Cell Commun Signal. 2024 Jan 12;22:35. doi: 10.1186/s12964-023-01455-z (PMC10785424; doi:10.1186/s12964-023-01455-z)
Supplement: Supplementary file 1 — Additional file 1. Full uncropped gels and blots. [file 12964_2023_1455_MOESM1_ESM.docx]

1. Hangyu Li, The Fourth Affiliated Hospital of China Medical University, Email: [sj__li_hangyu@sina.com](mailto:sj__li_hangyu@sina.com)
2. Hui Wu, First Hospital of Jilin University, Engaged in tumor immunology research, Email: [wuhui707@jlu.edu.cn](mailto:wuhui707@jlu.edu.cn)
3. David D Schlaepfer, Moores University of California San Diego (UCSD) Cancer Center, Email: [schlaepferdd@gmail.com](mailto:SchlaepferDD@gmail.com)
4. Tyler Jacks, Massachusetts Institute of Technology, Email: [tjacks@mit.edu](mailto:tjacks@mit.edu)
5. Brad H Nelson，University of British Columbia，Email: bnelson@bccrc.ca
